# Comparing the rates of methane production in patients with and without appendectomy: results from a large-scale cohort

**DOI:** 10.1038/s41598-020-57662-y

**Published:** 2020-01-21

**Authors:** Will Takakura, Sun Jung Oh, Tahli Singer-Englar, James Mirocha, Gabriela Leite, Adie Fridman, Mark Pimentel, Ruchi Mathur, Nipaporn Pichetshote, Ali Rezaie

**Affiliations:** 1 Division of General Internal Medicine, Department of Medicine, Cedars-Sinai, Los Angeles, 90048 USA; 2 Medically Associated Science and Technology (MAST) program, Department of Medicine, Cedars-Sinai, Los Angeles, 90048 USA; 3 Biostatistics and Bioinformatics Research Center, Cedars-Sinai, Los Angeles, 90048 USA; 4 Research Informatic and Scientific Computing Core Cedars-Sinai, Los Angeles, 90048 USA

**Keywords:** Archaeal biology, Clinical microbiology, Dysbiosis

## Abstract

There is no clear study identifying the microbiome of the appendix. However, in other diverticular conditions, such as diverticulosis, methanogens appear important. We investigated whether patients who had undergone appendectomies had decreased levels of exhaled methane (CH_4_). Consecutive patients who underwent breath testing (BT) from November 2005 to October 2013 were deterministically linked to electronic health records. The numbers of patients with CH_4_ ≥ 1 ppm (detectable) and ≥ 3 and ≥ 10 ppm (excess) were compared between patients who did and did not undergo appendectomy using a multivariable model adjusted for age and sex. Of the 4977 included patients (48.0 ± 18.4 years, 30.1% male), 1303 (26.2%) had CH_4_ ≥ 10 ppm, and 193 (3.9%) had undergone appendectomy. Appendectomy was associated with decreased odds of CH_4_ ≥ 1, ≥ 3, and ≥ 10 ppm (ORs (95% CI) = 0.67 (0.47–0.93), *p* = 0.02; 0.65 (0.46–0.92), *p* = 0.01; and 0.66 (0.46–0.93), *p* = 0.02, respectively). Additionally, the percentage of CH_4_ producers increased 4-fold from the first to ninth decade of life. This is the first study to report that appendectomy is associated with decreased exhaled CH_4_. The appendix may play an active physiologic role as a reservoir of methanogens.

## Introduction

The appendix is a narrow vermiform organ connected to the caecum. In humans, it can become inflamed and can be surgically removed without any overt consequences; therefore, it is often thought to be a vestigial organ. Some studies have cast doubt on this theory, arguing its potential role in immune function and maintenance of the gut microbiome. Its immunologic role is evidenced by histologic studies showing that the appendix houses large amounts of lymphoid tissues with both active T cells and B cells^1^. It is also a relatively rich source for IgA, and its activity seems to be maintained well into adulthood^2^. Moreover, the appendix contains a thick biofilm^3^, in which an abundance of microbes live^4,5^. The location and unique shape of the appendix makes it an ideal organ in which commensal organisms can be housed to repopulate the colon after the colonic gut microbiome has been modulated during diarrhoeal illnesses. However, to date, appendectomy has not been linked with an objective and clinically relevant microbial change.

Archaea are a unique group of microbes that share some features of bacteria (single circular chromosomes that lack introns with similar post-transcriptional modifications) and eukaryotes (use of histones in DNA packing and similar DNA replication, transcription, and translation mechanics)^6^. Most archaea in the human gut have a unique metabolic role in that they produce methane (CH_4_) as the end-product of their metabolism^7^. Most reduce carbon dioxide in the presence of hydrogen (H_2_) to produce CH_4_^8^. Two strict anaerobic strains of methanogens have been described in the human gut: *Methanosphaera stadtmaniae*^9^ and *Methanobrevibacter smithii*^10^. Methanogens have been associated and/or implicated in numerous human diseases, such as obesity, anorexia, constipation-predominant irritable bowel syndrome, periodontitis, and diverticulosis^11^.

In the clinical setting, breath testing (BT) is utilized to indirectly measure concentrations of CH_4_ and H_2_ produced in the gut^12^. As seen in humans and germ-free animal models, both gases are exclusively produced by the gut microbiota^13,14^. A subsequent study confirmed that patients who do not produce CH_4_ according to BT do not produce CH_4_ in their faeces^15^. Furthermore, antibiotics decrease CH_4_ levels, which seems to correlate with improvement in constipation associated with excessive CH_4_ production^16,17^.

Given the potential role of the appendix in the gut microbial population, we investigated the presence of CH_4_ in BTs in patients with and without an appendix.

## Results

A total of 10,967 patients were successfully linked to electronic health records. After including only those with elevated CH_4_ and normal BT, 4,977 patients were included in the final cohort. Of these, 193 underwent appendectomy before BT, and 4,784 patients retained their appendix at the time of BT. The mean ± SD age of patients at the time of BT was 48.0 ± 18.4 years and ranged from 2–101 years old. There was female predominance, with 1,496 (30.1%) males in the entire cohort. The rest of the demographics are shown in Table 1.

**Table 1:** Baseline Characteristics.

Variable		Study Cohort (n = 4977)	Appendectomy (n = 193)	No Appendectomy (n = 4784)
Age (years)		48.0 ± 18.4	58.4 ± 17.4	47.5 ± 18.3
Male		1496 (30.1%)	54 (28.0%)	1442 (30.1%)
BMI* (kg/m^2^)		24.8 ± 5.4	25.8 ± 5.8	24.7 ± 5.3
Race**	White	2866 (90.6%)	169 (90.4%)	2697 (90.6%)
	Black or African American	131 (4.1%)	8 (4.3%)	123 (4.1%)
	Asian	84 (2.7%)	4 (2.1%)	80 (2.7%)
	Other	82 (2.6%)	6 (3.2%)	76 (2.6%)

Patients with an appendectomy were older and more likely to be female than those without an appendectomy. BMI was higher in the group with appendectomy and race did not differ between the two groups. Values expressed as mean ± standard deviation or number (%). BMI = body mass index. *n = 2728, 181, 2547 for study cohort, appendectomy, and no appendectomy, respectively, due to missing data. **n = 3163, 187, 2976 for study cohort, appendectomy, and no appendectomy, respectively, due to missing data.

### Effect of age on CH_4_ production

**Figure 1:**
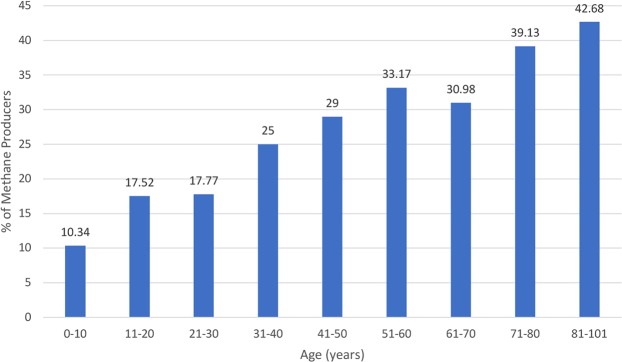
Depicts % of patients who had CH_4_ ≥ 1 ppm on their breath tests with age divided in increments of 10 years. The graph shows an increase in the % of patients who produced CH_4_ with age. CH_4_ = methane. n in each age group starting from 0–10 years was 58, 274, 698, 832, 962, 823, 736, 437, 157).

As previously described by our group, increased age was associated with an increased rate of excess CH_4_ production^18^. The percentage of patients with detectable CH_4_ increases with age, at 10.34% in those 0–10 years, 17.52% in those 11–20 years, 17.77% in those 21–30 years, 25% in those 31–40 years, 29% in those 41–50 years, 33.17% in those 51–60, 30.98% in those 61–79 years, 39.13% in those 71–80 years, and 42.68% in those 81–101 years (Fig. 1). For every 5-year increase in age, maximum CH_4_, area under the curve for CH_4_ (CH_4_ AUC), and baseline CH_4_ increased by 1.05 parts per million (ppm) (*p* < 0.0001), 5.20 ppm (*p* < 0.0001), and 0.55 ppm (*p* < 0.0001), respectively. In our multivariable model fitted for age, sex, and appendectomy (Table 2), for every 5-year increase in age, the odds of having CH_4_ levels above the threshold increased with odds ratios (ORs) (95% CI) of 1.10 (1.08–1.12) (*p* < 0.0001), 1.10 (1.08–1.12) (*p < *0.0001), 1.10 (1.08–1.12) (*p* < 0.0001) for CH_4_ ≥ 1 ppm, CH_4_ ≥ 3 ppm, and CH_4_ ≥ 10 ppm, respectively. In the multivariable linear regression model for those with detectable CH_4_, a 5-year increase in age was associated with an increase in CH_4_ AUC (95% CI) of 5.27 (3.47–7.06) ppm (*p* < 0.0001) and an increase in CH_4_ Max of 0.86 (0.52–1.20) ppm (*p* < 0.0001). For those with excess CH_4_, age was associated with a significant increase in the CH_4_ AUC of 5.10 (3.32–6.89) ppm (*p* < 0.0001) and 5.13 (3.40–6.86) ppm (*p* < 0.0001) for those with CH_4_ ≥ 3 ppm and CH_4_ ≥ 10 ppm, respectively. Similarly, the CH_4_ Max (95% CI) increased by 0.83 (0.49–1.16) ppm (*p* < 0.0001) and 0.82 (0.50–1.14) ppm (*p* < 0.0001) in the respective groups.

**Table 2:** Multivariable Logistic Regression Analysis (n = 4977).

Outcome	Variables	OR (95% CI)	P-value
CH_4_ ≥ 1 ppm	Appendectomy	0.67 (0.47–0.93)	0.02
	Age (5 years)	1.10 (1.08–1.12)	<0.0001
	Male	1.10 (0.96–1.26)	0.16
CH_4_ ≥ 3 ppm	Appendectomy	0.65 (0.46–0.92)	0.01
	Age (5 years)	1.10 (1.08–1.12)	<0.0001
	Male	1.10 (0.96–1.26)	0.15
CH_4_ ≥ 10 ppm	Appendectomy	0.66 (0.46–0.93)	0.02
	Age (5 years)	1.10 (1.08–1.12)	<0.0001
	Male	1.12 (0.97–1.28)	0.12

Appendectomy decreased the probability of having a detectable level or excess level of CH_4_. Increased age was significantly associated with CH_4_ production where as sex was not. CH_4_ = methane, ppm = parts per million.

### Effect of appendectomy on the odds and magnitude of CH_4_ production

According to the multivariable analysis adjusted for age and sex, those who underwent appendectomy were less likely to be CH_4_ producers or have pathological levels of CH_4_ (Table 2). Patients who had undergone appendectomy had an OR of 0.67 (0.47–0.93) (*p* = 0.02) for detectable CH_4_ ≥ 1 ppm. Sex was not a significant variable in this model, while age, as noted above, was significantly associated with increased odds of CH_4_ production. In the linear regression model of those who produced CH_4_ ≥ 1 ppm, appendectomy was not associated with a decreased magnitude of the CH_4_ AUC (Table 3) or CH_4_ Max (Table 4). Male sex was associated with a decreased CH_4_ AUC and CH_4_ Max, with coefficients (SD) of −30.53 (−43.99–−17.06) (*p* < 0.0001) and −4.91 (−7.44–−2.38) (*p* = 0.0001).

**Table 3:** Multivariable Linear Regression Analysis for CH_4_ AUC Above Cutoff Values.

Cutoff Values	Variables	Coefficient (95% CI)	P-value
CH_4_ ≥ 1 ppm (n = 1404)	Appendectomy	−3.50 (−38.34–31.34)	0.84
	Age (5 years)	5.27 (3.47–7.06)	<0.0001
	Male	−30.53 (−43.99– −17.06)	<0.0001
CH_4_ ≥ 3 ppm (n = 1393)	Appendectomy	−0.55 (−35.47–34.37)	0.98
	Age (5 years)	5.10 (3.32–6.89)	<0.0001
	Male	−30.91 (−44.31– −17.51)	<0.0001
CH_4_ ≥ 10 ppm (n = 1303)	Appendectomy	2.18 (−31.44–35.79)	0.9
	Age (5 years)	5.13 (3.40–6.86)	<0.0001
	Male	−35.13 (−48.01– −22.24)	<0.0001

Analysis of the magnitude of CH_4_ AUC in those with detectable and excess CH_4_. Appendectomy, age, and sex was modeled against patients who had CH_4_ ≥ 1, 3, or 10 ppm. Patients with CH_4_ less than the respective values were excluded. Appendectomy was not associated with an increase in CH_4_. Increased age and female sex were significantly associated with the magnitude of CH_4_ AUC. CH_4_ = methane, ppm = parts per million, AUC = area under the curve.

**Table 4:** Multivariable Linear Regression Analysis for CH_4_ Max Above Cutoff Values.

Cutoff Values	Variables	Coefficient (95% CI)	P-value
CH_4_ ≥ 1 ppm (n = 1399)	Appendectomy	0.26 (−6.27–6.80)	0.94
	Age (5 years)	0.86 (0.52–1.20)	<0.0001
	Male	−4.91 (−7.44– −2.38)	0.0001
CH_4_ ≥ 3 ppm (n = 1388)	Appendectomy	0.87 (−5.67–7.41)	0.79
	Age (5 years)	0.83 (0.49–1.16)	<0.0001
	Male	−4.98 (−7.49– −2.46)	0.0001
CH_4_ ≥ 10 ppm (n = 1298)	Appendectomy	1.49 (−4.72–7.69)	0.64
	Age (5 years)	0.82 (0.50–1.14)	<0.0001
	Male	−5.70 (−8.09– −3.32)	<0.0001

Analysis of the magnitude of CH_4_ max in those with detectable and excess CH_4_. Appendectomy, age, and sex was modeled against patients who had CH_4_ ≥ 1, 3, or 10 ppm. Patients with CH_4_ less than the respective values were excluded. Appendectomy was not associated with an increase in CH_4_. Increased age and female sex were significantly associated with the magnitude of CH_4_ max. CH_4_ = methane, ppm = parts per million.

Similarly, for CH_4_ ≥ 3 ppm and CH_4_ ≥ 10 ppm, patients who had undergone appendectomy had decreased odds of excess CH_4_, with ORs of 0.65 (0.46–0.92) (*p* = 0.01) and 0.66 (0.46–0.93) (*p* = 0.02), respectively (Table 2). Sex was not a statistically significant variable in the model. Similar to our linear regression analysis of CH_4_ ≥ 1 ppm, appendectomy did not seem to be associated with a change in the magnitude of excess CH_4_ Max or the CH_4_ AUC for those already producing CH_4_ ≥ 3 ppm and CH_4_ ≥ 10 ppm (Tables 3 and 4). Male sex was associated with a significant decrease in the CH_4_ AUC, with coefficients (95% CI) of −30.91 (−44.31–−17.51) (*p* < 0.0001) for CH_4_ ≥ 3 ppm and −35.13 (−48.01 –−22.24) (*p* < 0.0001) for CH_4_ ≥ 10 ppm; the CH_4_ Max coefficients (95% CI) were −4.98 (−7.49–−2.46) (*p* = 0.0001) for CH_4_ ≥ 3 ppm and −5.70 (−8.09–−3.32) (*p* < 0.0001) for CH_4_ ≥ 10 ppm.

In our study cohort, the area under the curve for H_2_ (H_2_ AUC) and baseline H_2_ levels did not differ between the appendectomy vs no appendectomy group, at 34.7 ± 26.0 ppm vs 36.6 ± 30.3 ppm (*p* = 0.57) and 2.9 ± 2.9 ppm vs 3.1 ± 3.7 ppm (*p* = 0.24), respectively.

## Discussion

When adjusted for age and sex, subjects with appendectomy were less likely to produce CH_4_. However, among those for whom CH_4_ was present, the magnitude of CH_4_ did not differ between the two groups, despite similar H_2_ levels. To our knowledge, this is the first study to show an association between appendectomy and a decreased rate of excess exhaled CH_4_.

It has been hypothesized that the appendix may serve as a reservoir for the gut microbiome due to its location and shape, making it relatively sheltered from microbial changes that occur in the rest of the colon^19,20^. During diarrhoeal illnesses, the appendix may function to repopulate the gut with its own luminal and mucosal microbiome^19^. Although causality cannot be establish, the theory that the appendix may act as a microbial reservoir is supported by our finding that the number of patients with detectable or excess CH_4_ was decreased in the appendectomy group, but the increase in magnitude in those for whom CH_4_ was present did not differ between appendectomy groups. Previous studies have shown that methanogens exist in the colonic walls and stool^21^, and given the notable difference in the surface area and volume of the colon vs the appendix, the bulk of CH_4_ production likely occurs in the rest of the gut as opposed to the appendix, which may act only as a reservoir. Interestingly, diverticulosis (a form of diverticula) has been linked to an increase in methanogens, and the appendix may have a similar function^22^. Although of note, the appendix, unlike the diverticulum, has a muscle layer and can perform antegrade peristalsis^23^; hence, it can potentially act as an active reservoir for the gut microbiome. Another potential explanation for the high rates of CH_4_ is that a particular composition of the microbiome is associated with appendectomies, and those who do not require surgery have increased amounts of CH_4_.

This association between CH_4_ and the appendix may have clinical implications. CH_4_ is associated with constipation, and studies have attempted to treat this based on eliminating methanogens. Non-systemic (i.e., poorly absorbed) oral antibiotic for the treatment of methanogenic archaea in the gut appears to have a high rate of recurrence in human subjects^24^. This phenomenon can be potentially be explained by the theory that the appendix serves as an active reservoir of methanogenic archaea with a thick biofilm resistant to antibiotic penetrance. Future studies should consider measuring the response and recurrence rates after antibiotic treatments between those with and without an appendix to determine whether the appendix is indeed acting as a reservoir.

Additionally, a history of appendectomy has been associated with a decreased risk for developing ulcerative colitis^25^ and an increased risk for Crohn’s disease^26^. In fact, appendectomy has been proposed as a potential treatment for ulcerative colitis^27^. In line with our hypothesis, the importance of the role of the appendix in inflammatory bowel diseases may in part be explained by the appendix acting as a reservoir to maintain the host gut microbiome.

Another interesting observation in this study was the association between methanogens (detected by the presence of CH_4_ in the breath) and age. Older patients are known to have increased CH_4_^28^ and since the prevalence of appendectomy increases with an individual’s age, this confounder warranted adjustment in this analysis. In addition, we found that age may be a significant contributor to increased CH_4_ levels. For every 5-year increase in age, there was approximately a 1 ppm increase in the CH_4_ Max, and there was a 4-fold increase in the percentage of CH_4_ producers from the patient’s first decade of life to the ninth decade of life (Fig. 1). This has been reported previously^18^. One possible explanation is that pockets in the intestine, such as the appendix and diverticula, may contribute to housing methanogens. This is evidenced by the fact that the prevalence of diverticulosis increases with age^29^. Alternatively, subjects with higher levels of methanogens may have increased archaeal compositions in the gut or have longer life expectancies than those with low levels of methanogens.

This study has several strengths and weaknesses. Given the referral status of the subjects, the results of the study may not be generalizable to the general population. Due to the retrospective design, we did not have detailed information regarding patients’ symptoms at the time of BT. Therefore, we were not able to correlate symptoms with the presence or absence of CH_4_ and appendectomy. Given that CH_4_ gas has been shown to decrease gut motility in humans and animal models^30^, it would be worthwhile to design a prospective study with symptom correlations. This new finding may hold clinical significance, as a reduction of CH_4_ levels has been shown to reduce constipation in humans^17^. There are several strengths to our study, including the large sample size and the use of the same fermentable sugar substrate (lactulose) with the same device.

In conclusion, there were decreased rates of CH_4_ in patients who had undergone appendectomy. Prospective studies measuring CH_4_ breath levels before and after appendectomy and correlating levels with symptoms, along with deep sequencing of the gut and appendix for methanogens, are warranted to investigate this new finding.

## Methods

### Subjects

Consecutive lactulose BT that was performed between November 2005 and October 2013 was analysed in this study. The breath tests were performed in patients referred to a tertiary care motility clinic by other providers. The research was approved by the Cedars-Sinai Internal Review Board (IRB Protocol 00034154) and completed in accordance with institutional regulations. All data analysed for this study, including BT results, were collected during routine clinical visits, and the IRB approved the use of the data without signed consent.

### Breath testing

All subjects consumed a special low-fermentable diet on the day before the test. Subjects were instructed to fast at least 12 hours prior to the test. All BT samples were collected at baseline and every subsequent 15 minutes for at least 2 hours after ingestion of 10 g of oral lactulose solution (Pharmaceutical Associates, Greenville, SC, USA). BT samples were analysed for H_2_ and CH_4_ after correction for carbon dioxide (CO_2_) levels using gas chromatography (Quintron Instrument Company, Milwaukee, WI, USA). CO_2_ levels were used to adjust H_2_ and CH_4_ levels to alveolar concentrations. Subjects with a normal BT results and subjects with elevated CH_4_ levels (≥10 ppm), as defined by the North American consensus statements, were included in the study. Subjects with elevated H_2_ levels (>20 ppm) and flatlines (non-CH_4_ and non-H_2_ producers) on the breath tests were excluded, as they may have indicated non-compliance with the diet, altered motility or hydrogen sulphide producers that competed for the H_2_ utilized by methanogens^31^.

### Data collection

Unique patient identifiers and deterministic record linkages were used to extract demographic data (age, sex, body mass index, and race) as well as appendectomy history. The appendectomy status and clinical history of patients were further confirmed by manual chart reviews. Patients were divided into two groups according to their history of appendectomy. If patients had undergone appendectomy before BT, they were included in the appendectomy group, whereas patients who had undergone appendectomy after BT were included in the no appendectomy group. Patients who we were not able to confirm the presence or absence of the appendix with respect to their BT date were excluded from the analysis.

### Statistical analysis

Numerical variables were summarized by means and standard deviations (SDs). Means of numerical variables with approximately normal distributions were compared across groups by independent samples t-tests. Categorical variables were summarized by frequencies and percentages, and group comparisons were made using chi-square tests. We defined the CH_4_ Max as the highest CH_4_ measured in one breath over the course of BT for every patient. The H_2_ AUC and CH_4_ AUC were calculated by the summation of H_2_ and CH_4_ levels at 90 minutes, respectively. Baseline CH_4_ and H_2_ were measured in the first breath prior to the administration of the lactulose. CH_4_ levels were markedly non-normal, with a high proportion of zero and small values, therefore they could not be fitted with standard linear regression. Thus, we assessed factors associated with CH_4_ levels in a two-step modelling procedure. First, we used multivariable logistic regression to model being at or above specific thresholds: ≥1 ppm (detectable) and the potentially clinically important thresholds of ≥3 ppm and ≥10 ppm (excess)^12^. In the second step, for those subjects at or above each specific threshold, we used multivariable linear regression to model the CH_4_ Max and CH_4_ AUC. To approximate a normal distribution, we excluded 5 outliers with the highest CH_4_ Max values in our analysis (this did not alter the significance of our study). Univariable comparisons of CH_4_ and H_2_ variables were made using Wilcoxon rank sum tests because of their highly skewed distributions. All analyses were performed using SAS version 9.4 (SAS Institute, Cary, NC, USA). We used a standard two-tailed alpha of 0.05 to determine significance.

## Data Availability

The datasets generated and analysed in the current study are available from the corresponding author upon reasonable request.
